# Potentiation of ^177^Lu-octreotate peptide receptor radionuclide therapy of human neuroendocrine tumor cells by PARP inhibitor

**DOI:** 10.18632/oncotarget.25266

**Published:** 2018-05-15

**Authors:** Nupur K. Purohit, Rashmi G. Shah, Samuel Adant, Michael Hoepfner, Girish M. Shah, Jean-Mathieu Beauregard

**Affiliations:** ^1^ Department of Molecular Biology, Medical Biochemistry and Pathology, Université Laval, Quebec City, Canada; ^2^ Cancer Research Center, Université Laval, Quebec City, Canada; ^3^ Neurosciences and Oncology Branches of CHU de Québec, Université Laval Research Center, Quebec City, Canada; ^4^ Department of Radiology and Nuclear Medicine, Université Laval, Quebec City, Canada; ^5^ Oncology Branch of CHU de Québec, Université Laval Research Center, Quebec City, Canada; ^6^ Institute of Physiology, Charité-Universitätsmedizin Berlin, Berlin, Germany

**Keywords:** peptide receptor radionuclide therapy, ^177^Lu-octreotate, neuroendocrine tumors, PARP inhibitor, radiosensitization

## Abstract

For patients with inoperable neuroendocrine tumors (NETs) expressing somatostatin receptors, peptide receptor radionuclide therapy (PRRT) with ^177^Lu-[DOTA0-Tyr3]-octreotate (^177^Lu-octreotate) is one of the most promising targeted therapeutic options but it rarely achieves cure. Therefore, different approaches are being tested to increase the efficacy of ^177^Lu-octreotate PRRT in NET patients. Using the gastroenteropancreatic BON-1 and the bronchopulmonary NCI-H727 as NET cell models, here we report that pharmacological inhibitors of DNA repair-associated enzyme poly(ADP-ribose) polymerase-1 (PARPi) potentiate the cytotoxic effect of ^177^Lu-octreotate on 2D monolayer and 3D spheroid models of these two types of NET cells. PARPi mediates this effect by enhancing ^177^Lu-octreotate-induced cell cycle arrest and cell death. Thus, the use of PARPi may offer a novel option for improving the therapeutic efficacy of ^177^Lu-octreotate PRRT of NETs.

## INTRODUCTION

Neuroendocrine tumors (NETs) originate from enterochromaffin cells of the diffuse neuroendocrine system and form a heterogeneous family of neoplasms. While NETs generally display an indolent behavior, they can significantly impair the quality of life, particularly when they cause hormonal syndromes [1]. About 68% of NETs arise from the gastroenteropancreatic system (GEP-NETs), and approximately 25% from the bronchopulmonary system (BP-NETs). In the last decade, their incidence has markedly increased by 6.4 fold, from 1.09 to 6.98 cases per 100,000 individuals per year [2–4]. Complete surgical resection remains the only cure, but it is not an option for all NET patients. Hence other treatment modalities, such as regional therapies, systemic chemotherapy, somatostatin analogues, interferon alpha, molecular targeted therapy and peptide receptor radionuclide therapy (PRRT) are employed [5]. Among these treatments, PRRT is one of the most promising in terms of survival, response rates and low toxicity [6]. PRRT uses radiolabeled somatostatin analogues, which preferably bind to somatostatin receptor (SSTR) subtypes 2 and 5 (SSTR2, SSTR5), which are overexpressed on the plasma membrane of NET cells [7, 8]. The ionizing radiations released by the particle-emitting PRRT radiopharmaceuticals cause cytotoxicity by inducing DNA damage, such as single and double strand breaks (SSBs and DSBs, respectively) [9]. To date, [^177^Lu-DOTA^0^,Tyr^3^]-octreotate (^177^Lu-octreotate) is the most widely used PRRT radiopharmaceutical and has shown favorable objective response rates, progression-free survival (PFS), overall survival and limited side effects [6, 8, 10]. However, complete remission following ^177^Lu-octreotate PRRT in patients with metastasized NET is still rare, and therefore there is a need for improvement. NETs are also sensitive to some cytotoxic and/or molecular targeted chemotherapies [11, 12], hence combining them with PRRT offers new possibilities for more effective treatments. Combination of capecitabine and temozolomide with ^177^Lu-octreotate has resulted in encouraging PFS with modest hematological toxicity in a clinical trial of progressive metastatic NETs [13]. Another trial of mTOR inhibitor everolimus along with ^177^Lu-octreotate is underway in NET patients [14].

Another class of molecularly targeted antitumor drugs are pharmacological inhibitors of poly(ADP-ribose) polymerase-1 (PARPi). These mediate their therapeutic effects by inhibiting the catalytic activity of the mammalian enzymes, poly(ADP-ribose) polymerases (PARPs), of which PARP1 is the most abundant member that accounts for about 80% of cellular PARP activity [15]. In mammalian cells, PARP1 is among the earliest proteins to detect and bind to different types of DNA damages, which results in its catalytic activation [16]. The activated PARP1 utilizes the substrate nicotinamide adenine dinucleotide (NAD^+^) to synthesize polymers of ADP-ribose (PAR) that post-translationally modify (PARylate) itself and other target proteins in the vicinity of DNA damage. The activation of PARP1 and PARylation of proteins have been shown to influence DNA damage responses such as DNA repair, chromatin remodeling and cell death [17]. Overall, PARP1 plays an important role in various types of DNA damage repair pathways including base excision repair of SSBs and homologous recombination repair (HRR) or nonhomologous end joining of DSBs [18]. Currently, the role of PARP1 in the base excision repair of DNA SSBs and abasic sites has been the basis for the use of PARPi as synthetic lethal monotherapy for BRCA1/2 mutant cancers that are defective in HRR pathway, or in combination with chemicals or external radiation for cancers with apparently normal DNA repair capacity [19–21].

With respect to the internal radiotherapy of cancer, PARPi was reported to potentiate radionuclide therapy using noradrenaline transporter-targeted radioiodinated metaiodobenzylguanidine in neuroblastoma cells [22]. A recent study has reported potentiation of ^177^Lu-octreotate PRRT by PARPi in a rat pancreatic adenocarcinoma cell line that endogenously expresses SSTR2, and in the human osteosarcoma cell line U2OS that was modified to express exogenous SSTR2 [23]. Since these are non-NET cancer models and expression of exogenous SSTR may not represent true pathophysiological response of NET cells that endogenously express SSTR, it still needs to be determined whether this effect of PARPi on ^177^Lu-octreotate-based PRRT will be observed in human NET cells. In the present study, we used 2D and 3D cell culture models of a human-derived GEP-NET and BP-NET cell lines, to show that PARPi potentiates therapeutic efficacy of ^177^Lu-octreotate PRRT in NET.

## RESULTS

### ^177^Lu-octreotate uptake and PARP1 activation in NET cells

**Figure 1:**
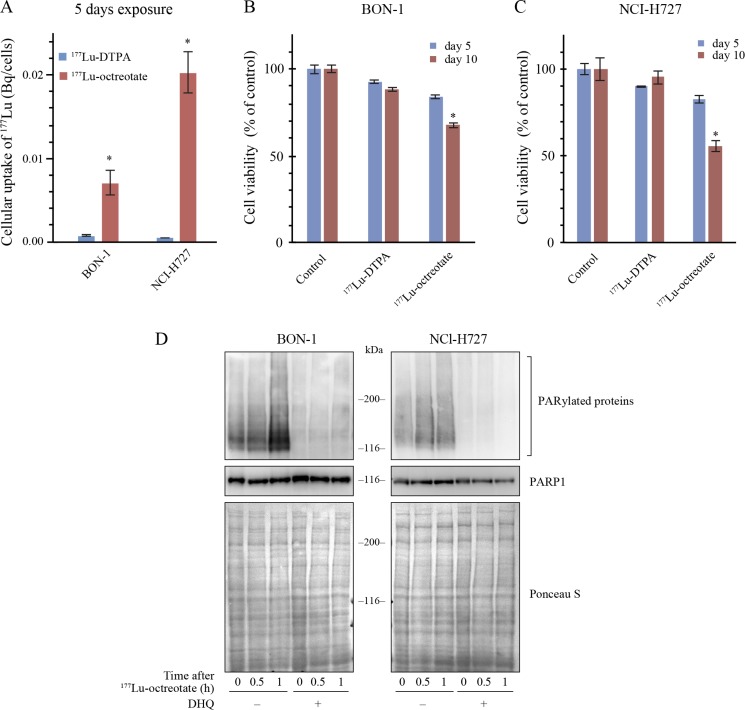
^177^Lu-octreotate uptake and PARylation of proteins in BON-1 and NCI-H727 cells (**A**) Uptake of ^177^Lu-octreotate in BON-1 and NCI-H727 cells. Both the cell lines were exposed to 2.75 MBq/mL of ^177^Lu-octreotate or 2.75 MBq/mL of ^177^Lu-DTPA for 5 days. Each data point, derived from six replicates per experimental condition, represents mean ± SEM. The ^*^ indicates significant differences (*P* ≤ 0.05) in uptake of ^177^Lu-octreotate as compared to that of ^177^Lu-DTPA in both the cell lines. (**B-C**) ^177^Lu-octreotate-induced reduction in cell viability of BON-1 and NCI-H727 cells. Both the cell lines were exposed to 2.75 MBq/mL of ^177^Lu-octreotate or 2.75 MBq/mL of ^177^Lu-DTPA for 5 days followed by five more days of incubation of cells in medium without radiolabel. The viability was determined at day 5 and day 10 of the protocol. The cell count in each treatment group is expressed as percent of number of viable cells in untreated control. The average of six replicates per experimental condition is plotted as mean ± SEM, with ^*^ indicating a significant difference in %viability of cells on day 5 and day 10 in each treatment group. (**D**) PARP inhibitor DHQ inhibits the PAR formation by PARP1 induced by ^177^Lu-octreotate in BON-1 and NCI-H727 cells. Both the cell lines were treated with 2.75 MBq/mL of ^177^Lu-octreotate in presence and absence of DHQ for indicated time points and the cell extracts were immunoblotted for PAR and PARP1.

We first screened a panel of selected NET and non-NET cell lines for the expression of mRNA of SSTR2 and SSTR5 by RT-PCR. Two human NET cell lines, BON-1 and NCI-H727 cells, were found to express both the receptors (Supplementary Figure 1A), confirming their reported receptor status [24, 25]. The functional state of these receptors was verified by comparing the intracellular uptake of two ^177^Lu radiochemicals, namely ^177^Lu-octreotate that is internalized via the SSTR and ^177^Lu-diethylenetriaminepentaacetic acid (^177^Lu-DTPA), a carrier of ^177^Lu that cannot penetrate inside the cell. There was a 5 to 20-fold increase in the intracellular uptake of ^177^Lu when these cells were incubated for five days with ^177^Lu-octreotate as compared to ^177^Lu-DTPA (Figure 1A), confirming the specificity of the SSTR-mediated internalization of ^177^Lu-octreotate. In BON-1 cells, although the intracellular uptake of ^177^Lu-octreotate was higher than ^177^Lu-DTPA even after 3h incubation (Supplementary Figure 1B), the 5-day incubation regime with ^177^Lu-octreotate resulted in a significantly higher toxicity as compared to 3h exposure, and this increased toxicity was not seen with ^177^Lu-DTPA (Supplementary Figure 1C). To assess whether the toxic effect was due to intracellular uptake of ^177^Lu-octreotate, we incubated both the cell lines for five more days after removal of each of the radiolabel at day 5 and compared the toxicity at 5th and 10th day (Figure 1B and 1C). There was a significant increase in toxicity of ^177^Lu-octreotate at day 10 as compared to day 5 in both BON-1 (Figure 1B) and H727 cells (Figure 1C). In contrast, a marginal toxicity attributable to external radiation by ^177^Lu-DTPA in the medium during 5-day exposure did not increase by day 10 (Figure 1B and 1C). Thus, the effect of ^177^Lu-octreotate was mostly attributable to its specific cellular internalization and intracellular retention rather than extracellular irradiation from ^177^Lu suspended in the medium.

Next, we examined the status of catalytic activation of PARP1 in response to DNA damage caused by irradiation from ^177^Lu-octreotate (Figure 1D). In both the cell lines, the immunoblotting of cell extracts up to 1 h after exposure to ^177^Lu-octreotate revealed a smear of heterogeneously PAR-modified proteins above 100 kDa up to 1 h. Moreover, the treatment with PARPi 1,5-dihydroxyisoquinoline (DHQ) before exposure to ^177^Lu-octreotate completely suppressed the signal of PAR in both the cell types. Our results indicate that the intracellular uptake of ^177^Lu-octreotate resulted in damage to DNA and PARylation of proteins that could be efficiently suppressed by PARPi; thus, PARPi has the potential to influence different cellular responses to radiation-induced DNA damage.

### Potentiation of ^177^Lu-octreotate by PARPi in BON-1 cell monolayers

**Figure 2:**
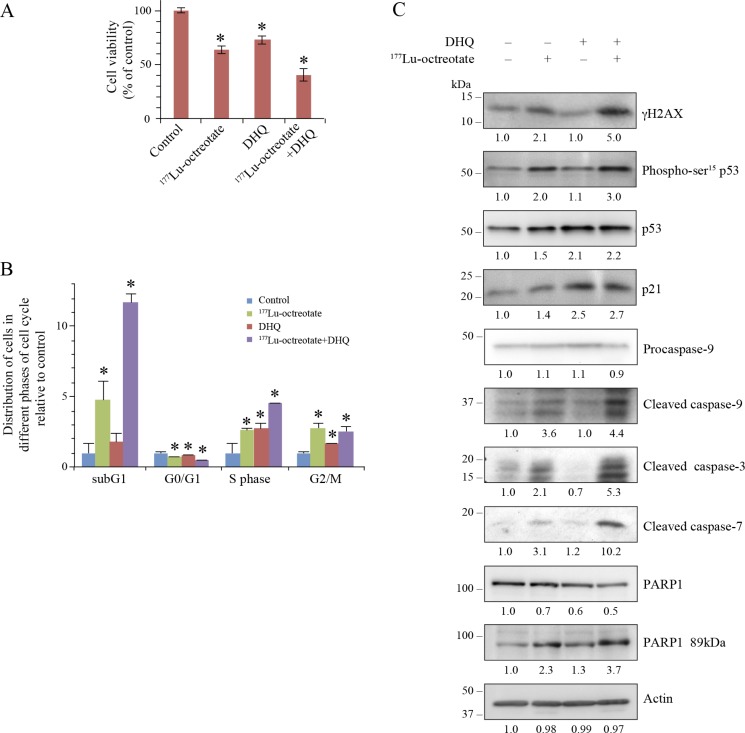
Effect of ^177^Lu-octreotate and PARPi on BON-1 cell monolayers (**A**) PARPi augments the ^177^Lu-octreotate-induced reduction in cell viability. The cells were treated in six replicates for five days with ^177^Lu-octreotate and DHQ independently and in combination followed by 10 more days of incubation of cells in medium without radiolabel and viable cell count was taken on the 10th day. The cell count is expressed as percent of viable cell count as compared to the untreated control. The number of cells seeded at the start of the experiment was 3.82% of the number of control cells on day of harvest. The average of six replicates per experimental condition are plotted as mean ± SEM and ^*^ indicates a significant difference from % viability of control cells. (**B**) PARPi potentiates ^177^Lu-octreotate-induced cell death and cell cycle arrest in BON-1 cells. The cells treated as above for panel-A were harvested at day 10 and analyzed by flow-cytometry after staining with propidium iodide. The data of each cell cycle phase (sub-G1, G1, S, and G2/M) is derived from triplicates per experimental condition and are plotted as mean ± SD of the fold change of the cell population relative to that of control of same phase of the cell cycle in the untreated controls and ^*^ indicates a significant difference from control cells in the given phase of cell cycle. (**C**) PARPi increases the downstream effects of ^177^Lu-octreotate–induced DNA damage on cell cycle arrest and apoptosis. The cells were treated in six replicates as indicated for panel A, harvested and pooled together for immunoblotting of parameters of DNA damage (γH2AX and phospho-p53), cell cycle arrest (p53 and p21) and apoptosis (Cleaved caspase 9, 3, 7 and PARP1 89kDa). Their band densities in arbitrary units were measured using GeneTools analysis software (Syngene) and normalized with the band density of actin from the corresponding samples. The fold change in the normalized band density of each protein in each treatment groups relative to control is indicated below each immunoblot. The panel for each immunoblot represents one of the two independent identical experiments with similar results.

We assessed the influence of suppression of PARP1 activation on the cytotoxic effect of ^177^Lu-octreotate in BON-1 cells using multiple parameters. Treatment with ^177^Lu-octreotate or DHQ alone reduced the fraction of viable cells to 63.4 % and 73.5 %, respectively, whereas these two agents together significantly reduced the viability to 40.4 % (Figure 2A). None of the treatments reduced the number of viable cells below the number of cells at the start of treatment, indicating growth-suppressive effect of the single or combination treatment. Moreover, this effect was due to radiolabel attached to octreotate because no toxicity was observed after treatment of cells with up to 200 nM unlabeled [DOTA^0^-Tyr^3^]-octreotate (Supplementary Figure 2A). The low-level cytotoxicity of PARPi observed with DHQ in BON-1 cells was also observed with two other PARPi: PJ-34 and ABT-888 (veliparib) (Supplementary Figure 2B). We also confirmed that treatment of BON-1 cells with the three different PARPi did not increase the intracellular uptake of ^177^Lu-octreotate (Supplementary Figure 2C). This indicates that the effect of PARPi, when combined with of ^177^Lu-octreotate was mainly due to its influence on biological events following intracellular irradiation.

To characterize the growth suppressive effect of ^177^Lu-octreotate and PARPi, we examined the proportion of cells in each phase of the cell cycle (Figure 2B and Supplementary Figure 3, left panel). ^177^Lu-octreotate treatment increased the cell population in sub-G1, S and G2/M phases by 4.8, 2.6 and 2.8 folds, respectively. Since sub-G1 phase represents apoptotic cells with reduced DNA content [26], these results indicate a combination of cell death and cell cycle arrest in S and G2/M phases in response to ^177^Lu-octreotate. While PARPi treatment alone did not cause a significant increase in sub-G1 population, its presence with ^177^Lu-octreotate significantly increased sub-G1 fraction of cells (12 folds), accompanied by an increased S-phase arrest (4.5 folds). Thus, ^177^Lu-octreotate induced apoptosis and cell cycle arrest, which were augmented by co-treatment with PARPi.

### Mechanisms of the potentiation of ^177^Lu-octreotate by PARPi in BON-1 cells

To examine the consequences of DNA damage induced by irradiation from ^177^Lu-octreotate, we assessed the phosphorylation status of histone H2AFX (γH2AX) and of TP53 (p53) [27]. Treatment with ^177^Lu-octreotate increased the levels of γH2AX and phospho-p53. While PARPi alone did not alter these parameters, it significantly increased the levels of ^177^Lu-octreotate-induced γH2AX and phospho-p53 (Figure 2C). The phosphorylation of p53 is known to promote its accrual [28], which was observed following treatment with ^177^Lu-octreotate, PARPi, and the combination of the two (Figure 2C). Furthermore, while ^177^Lu-octreotate alone caused a modest upregulation of the cell cycle inhibitor CDKN1A (p21), PARPi alone or in combination with ^177^Lu-octreotate caused a significant increase in p21 levels. These results demonstrate the effect of PARPi in causing the persistence of damaged DNA and cell cycle arrest.

The increased sub-G1- population of ^177^Lu-octreotate-treated cells with or without PARPi indicates an involvement of apoptosis [29]. The immunoblotting revealed that all three caspases 3, 7 and 9 were significantly activated after treatment with ^177^Lu-octreotate, but not with PARPi (Figure 2C). However, the presence of PARPi further upregulated ^177^Lu-octreotate-induced activation of all three caspases. There was a corresponding increase in cleavage of PARP1 to its signature 89-kDa fragment by caspases 3 and 7 in these samples (Figure 2C). Together, the changes in parameters that assess cellular responses to DNA damage, stalled stalled cell cycle (increased p21), and cell death (subG1 cells, and activation of caspases), indicate that PARPi potentiated cytotoxicity of ^177^Lu-octreotate in BON-1 cells by upregulating cell cycle arrest and apoptosis.

### Potentiation of ^177^Lu-octreotate by PARPi in BON-1 cell spheroids

**Figure 3:**
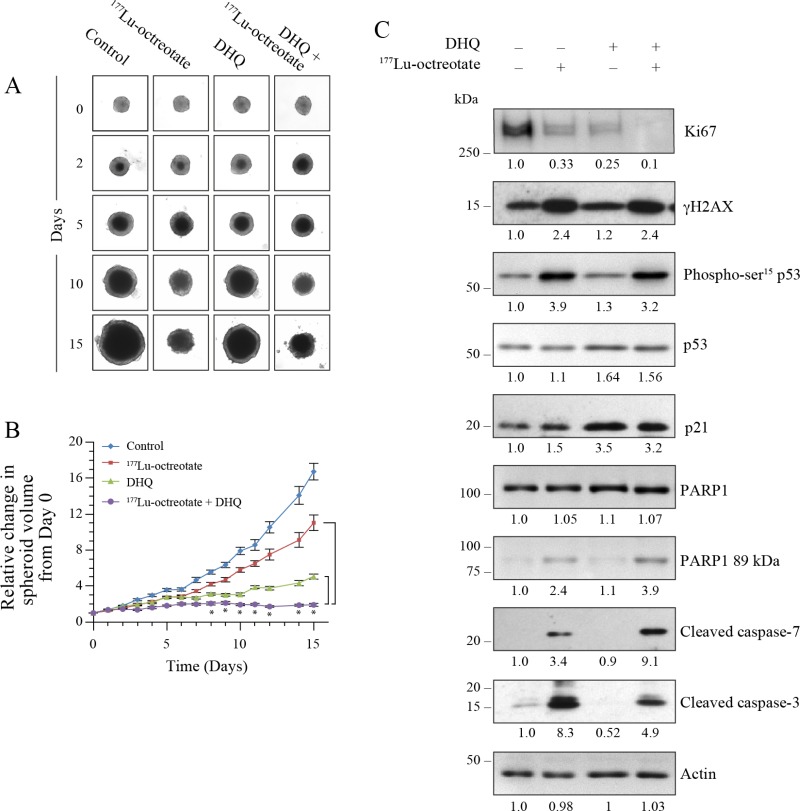
Effect of ^177^Lu-octreotate and PARPi on BON-1 spheroids (**A**) The 12 control and 24 treatment spheroids of BON-1 cells were treated with ^177^Lu-octreotate and DHQ independently and in combination for 5 days followed by 10 more days of incubation of cells in medium without radiolabel. The representative image of 12 to 24 spheroids over time course of treatment from two independent experiments is shown here. The black core and the peripheral grey zones represent dead and viable parts of the spheroid, respectively. (**B**) The data pooled from time-course of changes in spheroid volume from 12 to 24 spheroids from two independent experiments described for panel A is plotted as mean ± SEM of fold change in the spheroid volume relative to that at the start of treatment, i.e. Day 0. ^*^ indicates that all the data points are significantly different between DHQ alone and ^177^Lu-octreotate + DHQ treatment groups as well as between ^177^Lu-octreotate and ^177^Lu-octreotate + DHQ treatment groups from each other from day 8 onwards with *P*-values ≤ 0.01. (**C**) Analyses of different parameters of cell proliferation, DNA damage, cell cycle arrest and apoptosis. The 12 control and 24 treatment spheroids were treated as indicated for panel A and harvested on day 15 for immunoblotting proliferation marker Ki67 and other indicated parameters and band intensities were measured and expressed for each panel as described for Figure 2C. The panel for each immunoblot represents one of the two independent identical experiments with similar results.

The 3D spheroids mimic physiological and biological properties of *in vivo* tumors better than 2D cell monolayers for testing anti-cancer therapeutics [30]. We examined the PARPi (DHQ)-mediated potentiation of cytotoxicity of ^177^Lu-octreotate in a 3D model of BON-1 cells (Figure 3A). We first confirmed the uptake of ^177^Lu-octreotate by BON-1 spheroids and noted that the presence of PARPi did not significantly change the extent of uptake of ^177^Lu-octreotate by spheroids (Supplementary Figure 4A). Next, we determined the effect of ^177^Lu-octreotate with or without PARPi on the growth of spheroids (Figure 3B). Over 15 days, while the volume of untreated spheroids increased by 16 folds (100%), the spheroids treated with ^177^Lu-octreotate, PARPi alone or ^177^Lu-octreotate plus PARPi increased by 5.0 (30%), 11.0 (70%) and 1.9 folds (12%), respectively. The reduction in spheroid growth with ^177^Lu-octreotate appeared specific to ^177^Lu because the unlabeled [DOTA^0^-Tyr^3^]-octreotate up to 200 nM had no effect on the growth of spheroids. (Supplementary Figure 4B). Thus, like in the 2D model, the combination of PARPi and ^177^Lu-octreotate was more effective than ^177^Lu-octreotate alone in suppressing the growth of 3D cultures of BON-1 cells *in vitro*.

The levels of cell proliferation marker MKI67 (Ki67) is an important indicator of tumor growth and aggressiveness used for grading NETs [31]. The immunoblotting revealed reduced levels of Ki67 in ^177^Lu-octreotate- or PARPi-treated spheroids as compared to untreated spheroids (Figure 3C). Once again, the combined treatment was most effective in reducing the Ki67 level as compared to the individual treatments. This is consistent with a stronger suppression of growth of BON-1 spheroids in the presence of ^177^Lu-octreotate plus PARPi.

In order to further understand the mechanisms by which ^177^Lu-octreotate and PARPi affected the spheroids growth, we examined DNA damage and cell death parameters 15 days after treatment (Figure 3C). The immunoblotting of spheroid extracts revealed that ^177^Lu-octreotate alone caused an induction of DNA damage markers γH2AX and phospho-p53. PARPi alone did not affect the phosphorylation of either marker but, in combination with ^177^Lu-octreotate, a pronounced increase was observed. PARPi treatment resulted in upregulation of p53 and p21 with and without ^177^Lu-octreotate. Regarding cell death parameters, ^177^Lu-octreotate by itself increased the cleaved caspase 3. The combined treatment resulted in higher amount of cleaved caspase 7 with a concomitant increase in PARP1 cleavage. In summary, the results with the 2D and 3D models of BON-1 cells indicate that ^177^Lu-octreotate suppressed cell growth, caused DNA damage, cell cycle arrest and cell death, and these effects were augmented in the presence of PARPi.

### Potentiation of ^177^Lu-octreotate by PARPi in H727 cell models

**Figure 4:**
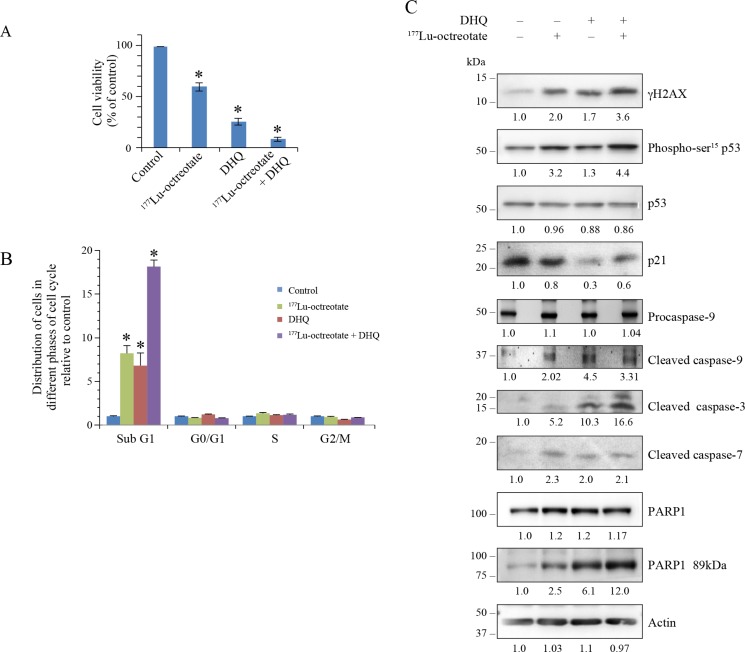
Effect of ^177^Lu-octreotate and PARPi on NCI-H727 cell monolayers (**A**) PARPi augments the ^177^Lu-octreotate-induced reduced cell viability of NCI-H727 cells. The H727 cells were treated in six replicates for five days with ^177^Lu-octreotate and DHQ independently and in combination as described for BON-1 cells in Figure 2. The average cell counts in each treatment groups from six independent experiments is derived and expressed as mean ± SEM exactly as described for Figure 2A. The number of cells seeded at the start of the experiment were 3.75% of the number of control cells on day of harvest. ^*^ indicates a significant difference from % viability of control cells. (**B**) PARPi potentiates the ^177^Lu-octreotate-induced cell death and cell cycle arrest in H727 cells. The cells treated as above for panel-A were harvested at day 10 and analyzed by flow-cytometry after staining with propidium iodide. The data of each cell cycle phase (sub-G1, G1, S, and G2/M) is derived from triplicates per experimental condition and were plotted as means ± SD of fold change in the percent cell population relative to that of untreated controls. The ^*^ indicates a significant difference as compared to the control cells in the given cell cycle phase. (**C**) Analyses of DNA damage, cell cycle arrest and cell death parameters by western blotting in H727 cells. The cells treated in six replicates as indicated for panel A were harvested and pooled together for immunoblotting of various parameters. Band intensities were measured as described for Figure 2C. The panel for each immunoblot represents one of the two independent identical experiments with similar results.

Using trypan blue dye exclusion assay, we observed that the growth of H727 monolayer cells was reduced to 59.4 % by ^177^Lu-octreotate and to 25.5 % by PARPi DHQ (Figure 4A). The combination treatment reduced the growth to 8.5 % of control. In comparison, the treatment of cells with unlabeled [DOTA^0^-Tyr^3^]-octreotate up to 200 nM did not result in any toxicity (Supplementary Figure 2D), confirming that the effect of ^177^Lu-octreotate was due to the internalized radiolabel. We confirmed the above results in H727 cells using two other PARP inhibitors: PJ-34 and ABT-888 (Supplementary Figure 2E). Moreover, as in BON-1 cells, the presence of PARPi did not affect the uptake of ^177^Lu-octreotate by H727 cells (Supplementary Figure 2F). The FACS analyses revealed that the growth reduction in these cells at 10 days under all treatment conditions largely manifested as cell death (Figure 4B and Supplementary Figure 3, right panel). The augmentation of population in sub-G1 phase in the presence of ^177^Lu-octreotate, PARPi, and ^177^Lu-octreotate plus PARPi was 8.2, 6.8 and 18.1 folds over the control, respectively. At the time of harvesting, none of the treatment conditions exhibited much difference in proportion of cells in other phases of cell cycle. The DNA damage parameters such as γH2AX and phospho-p53 increased in the presence of ^177^Lu-octreotate or PARPi alone, and there was an additive effect following the combined treatment (Figure 4C and 4D). Conversely, levels of p53 remained unaffected by any of the three treatments and PARPi lowered the levels of p21 in these cells. Since we observed a significant percentage of H727 cells in sub-G1 phase which represents apoptotic population of the cells [26], we also verified the markers of apoptosis in these cells. We observed that in the three treatment groups, there was an activation of caspase 9 as compared to control (Figure 4C and 4D). Moreover, both the downstream caspases 3 and 7 were activated in H727 cells. PARPi caused an increase in cleaved caspase 3, demonstrating its toxicity in these cells, but the combined treatment yielded much higher levels of activated caspase 3 than single treatment. The cleavage of PARP1 to its 89-kDa fragment correlated with caspase 3 activation in these cells.

**Figure 5:**
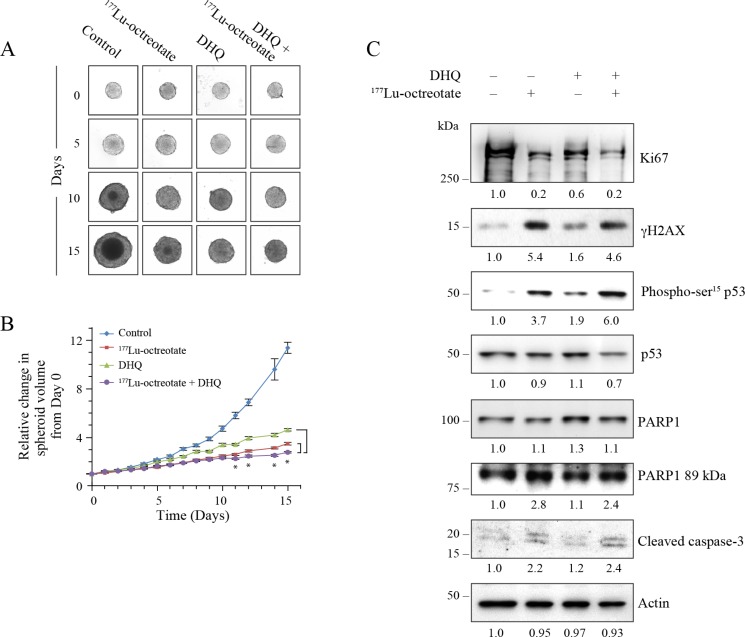
Effect of ^177^Lu-octreotate and PARPi on NCI-H727 cell spheroids All three panels represent data from H727 spheroids exactly treated as described for BON-1 spheroids in Figure 3. (**A**) The representative microscopic images of spheroids from each treatment group at indicated time points. (**B**) The time-course of change in spheroid volume from two independent experiments described for panel A is plotted as mean ± SEM of fold change in the spheroid volume relative to that at the start of treatment, i.e. Day 0. ^*^ indicates that all the data points are significantly different between DHQ alone and ^177^Lu-octreotate + DHQ treatment groups as well as between ^177^Lu-octreotate and ^177^Lu-octreotate + DHQ treatment groups from each other from day 8 onwards with *P*-values ≤ 0.01. (**C**) Analysis of cell proliferation marker Ki-67, DNA damage, cell cycle arrest and apoptosis parameters by western blotting. The treatments, immunoblotting and data analyses were carried out as described for BON-1 spheroids in Figure 3C.

In the 3D spheroid model of H727 cells, we first confirmed the uptake of ^177^Lu-octreotate (Supplementary Figure 4C). There was a trend toward higher uptake of ^177^Lu-octreotate in the presence of PARPi, but this was not statistically significant. The control spheroids exhibited a 11.4-fold (100%) growth over 15 days, whereas those treated with ^177^Lu-octreotate, PARPi, and ^177^Lu-octreotate + PARPi grew by, 4.6 (23%), 3.5 (30%) and 2.8 folds (18%), respectively (Figure 5A and 5B). Unlabeled [DOTA^0^-Tyr^3^]-octreotate by itself had no effect on the growth of spheroids (Supplementary Figure 3D). ^177^Lu-octreotate and PARPi caused a reduction of Ki67 level, which was further reduced with the combination treatment (Figure 5C and 5D). γH2AX and phospho-p53 were increased with ^177^Lu-octreotate alone, but not with PARPi alone, while the combination treatment yielded levels comparable to those following ^177^Lu-octreotate alone (Figure 5C). Like in the 2D monolayers, p53 levels remained unchanged in the 3D model. ^177^Lu-octreotate by itself caused some activation of caspase 3, and the combined treatment further enhanced it. We observed PARP1 cleavage in all the four groups, but it was higher in the spheroids treated with ^177^Lu-octreotate and ^177^Lu-octreotate plus PARPi. Collectively, the results of the 2D and the 3D models of H727 cells indicated that PARPi alone was generally toxic by itself, and it further increased inherent capacity of ^177^Lu-octreotate to cause cell death.

## DISCUSSION

^177^Lu-octreotate PRRT, when coupled with molecular imaging of SSTR, is one of the most promising theranostic applications for the treatment of inoperable NETs. Different approaches are being investigated to improve the antitumor efficacy of ^177^Lu-octreotate PRRT and one of them is combining it with radiosensitizing chemotherapeutic drugs [13, 14, 32]. In the present study, we utilized NET patient-derived GEP-NET and BP-NET cell lines that endogenously express SSTR2 and 5, to show that ^177^Lu-octreotate is internalized by these cells and results in PARylation of proteins indicating the catalytic activation of PARP1 in response to DNA damage caused by internalized ^177^Lu-octreotate. We also demonstrated that the pharmacological suppression of PARP-activation response results in a potentiation of cytotoxic effects of ^177^Lu-octreotate. We also show that PARPi did not increase uptake of ^177^Lu-octreotate by the cells, but augmented consequences of radiation-induced damage to DNA, such as blocked cell cycle, increased signal for γH2AX or phosphorylated p53. These observations are in agreement with known biological function of PARP1 activation in facilitating the downstream processes such as the repair of DNA damage [17]. More specifically, PARPi has been shown to inhibit repair of DNA damage via trapping PARP1 at the lesion site and convert unrepaired SSBs to DSBs leading to cell death [21, 33]. Our data is in agreement with a recent report that PARPi increases the signal for TP53BP1, a marker of DSB, in the biopsy tissues of NET patients treated with ^177^Lu-octreotate [23].

The increased DNA damage is known to result in the phosphorylation-induced activation and accumulation of p53 via the ATM, ATR and DNA PKcs pathways [27, 28], which can lead to cell cycle arrest and/or apoptosis [34]. Our data suggest that p53 accumulation and its phosphorylation could be the reason for the observed cell cycle arrest and apoptosis in GEP-NET BON-1 cells. In contrast, in BP-NET H727 cells, there was increased phosphorylation of p53 but not its accumulation, which may be due to mutations reported in p53 gene in H727 cells [35, 36]. The lack of p53 or p21 accumulation in H727 cells may also be due to the greater tendency to execute apoptosis rather than remain stalled in cell cycle. The apoptotic cell death by PARPi and ^177^Lu-octreotate treatment alone or in combination in H727 and BON-1 cells was evident from reduced cell viability and the presence of activated (cleaved) caspases 9, 3 and 7, and apoptosis signature cleavage of PARP1 to its 89-kDa fragment. Our results with these two NET cell lines suggest that PARPi could potentiate the cytotoxic responses to ^177^Lu-octroetate in different NET tumors, which have normal or abnormal p53/p21 response to DNA damage.

We also observed that treatment of NCI-H727 cells with PARPi alone induced a relatively high level of cytotoxicity as compared to that seen in BON-1 cells, which could be due to the presence of missense mutations in BRCA1 gene in these cells [37]. In fact, PARPi are used in clinic as synthetic lethal monotherapy for BRCA-mutant ovarian tumors that are deficient in HRR of DSBs [19], although it needs to be confirmed whether the BRCA1 mutation affects the functional state of BRCA in H727 cells. In any case, ^177^Lu-octreotate with PARPi caused more damage in BON-1 and NCI-H727 cells than either compound alone, indicating a collaborative action of these two agents. Thus, our studies strengthen the argument that PARPi can potentiate the therapeutic efficacy of DNA damaging agents, even in BON-1 cells that are not characterized by BRCAness, i.e. any known deficiency in DNA DSB repair.

Radiosensitization by PARPi during the ^177^Lu-octreotate-based PRRT of osteosarcoma cell line U2OS expressing exogenous SSTR2 and of rat pancreatic tumor cell line Ca20948 expressing endogenous SSTR2 has been recently reported [23]. However, osteosarcomas are not clinically treated with PRRT as they do not express SSTR, and artificially overexpressed SSTR may not reflect pathophysiological conditions in these cells. Further, the biology of osteosarcoma or of a rat pancreatic cancer of acinar origin [38] differs from that of NETs in many respects, as they do not have the same origin. In contrast, PRRT with ^177^Lu-octreotate has become a standard therapeutic option for endogenously SSTR-expressing NETs in the clinic [39]. While the authors in this study clearly showed radiosensitization by PARPi of PRRT in SSTR-positive cells, our results now firmly establish that PARPi has the capacity to potentiate ^177^Lu-octreotate-based PRRT in two human NET cell lines from common origins: gastroenteropancreatic and bronchopulmonary.

It may be challenging to directly translate the therapeutic effects seen with 2D monolayer cells to 3D tumors *in vivo,* which are comprised of a variety of cells that interact with each other, have a heterogeneous distribution of receptors on their surface, have diffusional limits to mass transport of drugs, nutrients and other factors, and may develop central necrosis and regions of hypoxia. These *in vivo* biological conditions can be partly recreated *in vitro* by using 3D spheroids of the cancer cell lines [30]. In our study, we showed that the radiosensitizing effects of PARPi observed in 3D spheroids were not only similar to those observed using monolayer culture (2D) of these cells, but also mediated via similar mechanisms of action. Moreover, the observed cytotoxic effects using PARPi and ^177^Lu-octreotate alone or in combination directly correlated with the levels of cell proliferation marker Ki-67. Thus, our data with 3D spheroids supports the use of PARPi during PRRT in pre-clinical animal models of NET, and eventually in clinical trials in NET patients.

PRRT confers an advantage of limited long-term toxicity or acute and sub-acute side effects owing to its specificity towards the SSTRs, which are more concentrated in the NET lesions as compared to healthy organs [6]. Therefore, it would be beneficial to combine PRRT with a potentiating agent that is also specific and associated with limited toxicity that is not overlapping with that of PRRT. PARPi is one such agent that is known to have a favorable toxicity profile [40, 41], and also causes its radiosensitizing or chemo-potentiation effects preferentially in high-grade metastatic cancers [15, 16]. This is because PARPi-mediated suppression of repair of SSBs and conversion to DSBs can take place only in replicating cells. Thus, in the tumors containing a higher proportion of replicating cells that are defective in cell cycle checkpoint responses than normal tissues, PARPi can increase the therapeutic index of PRRT by specifically increasing DNA damage in actively replicating NET cells, while sparing non-cycling normal tissues. Hence, PARPi is not expected to potentiate ^177^Lu-octreotate effects in organs with high radiation exposure but with lower proliferative activity, such as the kidney. However, some side effects could still potentially occur in the bone marrow, even if it receives relatively low absorbed radiation doses during PRRT, because of its high proliferation rate and radiosensitivity.

In summary, our study shows that PARPi potentiates PRRT in human NET cell lines via augmenting the downstream effect of ^177^Lu-octreotate-induced DNA damage, such as cell cycle arrest and apoptosis. Since some PARP inhibitors such as olaparib are being used in the clinic for the treatment of other cancers, combining PARPi with ^177^Lu-octreotate could rapidly offer a new opportunity for boosting the efficacy of PRRT in patients suffering from NET.

## MATERIALS AND METHODS

### Chemicals and other reagents

The chemicals used in preparing the buffers and other fine chemicals were purchased from Sigma. All the cell culture-related products were purchased from Life technology. Nitrocellulose ECL membrane was from Amersham and immobilon western chemiluminescent HRP substrate (WBKLS0500) was from Millipore. PARP inhibitors, 1,5-dihydroxyisoquinoline (DHQ) was from Sigma, PJ-34 was from Alexis Biochemicals and ABT-888 (veliparib) was from Santa Cruz Biotechnology.

### Radiopharmaceuticals

^177^Lu-octreotate radiolabeling was performed as previously described [42] and was used for clinical PRRT as well as for all the experiments. ^177^LuCl_3_ was obtained from IDB Holland BV, and [DOTA^0^,Tyr^3^]-octreotate was generously provided by the Erasmus Medical Center (Rotterdam, The Netherlands). Radiochemical purity of ^177^Lu-octreotate was >97%, and specific activities in different batches ranged from 22,015 to 85,100 MBq/µmole (50–100 nM octreotate delivered per 2.75 MBq typical treatment dose). The ^177^Lu-DTPA was prepared by mixing ^177^LuCl_3_ with DTPA at room temperature.

### Cell culture and treatment

The BON-1 is a GEP-NET cell line established from a human pancreatic carcinoid tumor [43] which was maintained as described earlier [44]. The BP-NET NCI-H727 cells (CRL-5815) were obtained from ATCC and maintained as per the ATCC specifications.

Unless specified otherwise in the legends, BON-1 and H727 cells were seeded in monolayers at 10,000 cells/cm^2^ and subjected to treatments after 2 days either individually or in combination with ^177^Lu-octreotate and one of the different PARP inhibitors, 100 µM DHQ, 10 µM PJ-34 or 2.5 µM ABT-888. For relevant treatment groups with PARPi, the cells were treated with PARPi starting 30 minutes prior to exposure to ^177^Lu-octreotate (2.75 MBq/mL) for five days. The medium was removed after five days and cells were maintained for five more days in the presence of PARPi in relevant treatment groups. Similar experimental conditions were also used for treatment of cells with ^177^Lu-DTPA individually, i.e. 5 days of treatment followed by the removal of treatment and maintaining cells for five more days.

### Growth of spheroids

The 3D spheroids of BON-1 and H727 cells were grown as previously described [45]. After six days, when the spheroids reached 300–400 µM diameter, they were treated with ^177^Lu-octreotate 2.75 MBq/mL medium (or mock) in the presence or absence of PARPi for five days. The treatment was terminated after five days with change of medium, and the spheroids were grown for an additional 10 days in the presence of PARPi, where required. The images of spheroids were captured at 20X magnification using Zeiss Axiovert 200 microscope and volume of spheroids was calculated (v = 0.5 × length × (width)^2^) from the dimensions measured using Axio Vision 4.9.1 Software. The average growth from six spheroids per treatment group up to 15 days was calculated as relative to the spheroid volume at the start of treatment (Day 0). For the analyses of ^177^Lu-octreotate uptake and protein analyses by immunoblotting, 12–24 spheroids were pooled per treatment group at 15 days.

### Measurement of ^177^Lu-octreotate uptake

Cell monolayers growing in 6-well clusters were incubated with ^177^Lu-octreotate and different PARP inhibitors for five days, as described above. The medium was removed and monolayers were washed thrice with phosphate buffered saline (PBS) to remove any unbound ^177^Lu-octreotate. Cells were scrapped in 1 mL PBS and 0.2 mL aliquot was mixed with 5 mL of scintillating liquid for measuring radioactivity by liquid scintillation counter (Coulter). Another aliquot of cells was used for viable cell count. ^177^Lu-octreotate uptake was presented as Bq per cell.

To measure ^177^Lu-octreotate uptake by spheroids, 6–12 spheroids were collected per treatment group in 100 µL culture medium on a Millipore glass-fiber filter with 0.7 µm retention under vacuum. After two washes with PBS, radioactivity was measured as mentioned above. ^177^Lu-octreotate uptake was presented as Bq/mm^3^ volume of spheroid.

### Cell viability by trypan blue

BON-1 and H727 cells in six-well clusters were treated with PARPi and ^177^Lu-octreotate as described before. Six replicates per treatment group and per time point were used. At the specified time points, cells were trypsinized to prepare single-cell suspension from which an aliquot was mixed with equal volume of 0.4 % trypan blue (GIBCO) to count viable cells which exclude the dye.

### Flow cytometric analysis of cell cycle

Cell monolayers of BON-1 and H727 treated as described above were trypsinized, washed twice and suspended in PBS. An aliquot representing one million cells from each sample was fixed with 70% ethanol on ice for 30 min and spun down at 500 *g* to remove ethanol. The pellet was washed twice with PBS, suspended in 500 µL of PBS containing 50 µg/mL propidium iodide and 50 µg/mL RNAase A and incubated at 37° C for 30 minutes. FACS analyses were carried out with a BD FACS Calibur flow cytometer, and the data were analyzed with BD FACSDiva software. The histograms were generated using FlowJo 7.6.1 software from Tree Star.

### Western blotting

Cell monolayers of BON-1 and H727 treated as described above were scrapped in PBS, spun down, suspended in 1X Laemmli SDS-PAGE buffer and sonicated to prepare protein extracts for SDS-PAGE. In case of spheroids, 6–12 spheroids per treatment groups were collected on Day 15 in 100 µL volume and pooled into one tube. The spheroids were spun down and washed with PBS and suspended in 1X Laemmli SDS-PAGE buffer and sonicated to prepare protein extracts for SDS-PAGE. 10–20 µg of protein extracts from each treatment groups of cell monolayers or spheroids were resolved on 6–15% gradient SDS-PAGE or on individual 8 and 15% SDS-PAGE, transferred to nitrocellulose membrane and probed with specific antibodies as indicated. The immunoprobing for β-actin or staining of blot with Ponceau S were performed as the loading control. All Western blotting data were repeated twice from the extracts derived from two independent experiments. Their band densities in arbitrary units were derived using GeneTools analysis software from Syngene. All the band densities of different proteins are normalized with the band density of actin from the corresponding samples.

### Antibodies

The following antibodies were used at specified dilutions for immunoblotting: polyclonal PARP1 antibody (1:5,000; Alexis Biochemicals), PARP1 monoclonal (F2, 1:500; Santacruz), PARP1 C-2-10 (1:1,000), PARP1 89kDa (1:1,000, Abcam), p53 (1:1,000, Boehringer), gH2AX (1:1,000, Millipore), phospho-p53 (1:2,000, NEB), p21 (1:1,000, NEB0), β-actin (1:20,000, Sigma), cleaved caspase-9 (1:1,000, Cell Signaling), cleaved caspase-3 (1:1,000, Cell Signaling), cleaved caspase-7 (1:200, Abcam), Ki-67 (1:100, Thermo scientific), PCNA (1:1,000, Neomarkers). Anti-pADPr monoclonal 10H was purified from the culture medium of 10H hybridoma obtained from Dr. M. Miwa, National Cancer Center Research Institute, Tokyo, through the Riken cell bank (1:500). The secondary HRP-conjugated antibodies (1:1,250) were purchased from Jackson Immunoresearch Laboratories.

### RT-PCR for SSTR2 and SSTR5

Total RNA was extracted from BON-1 and NCI-H727 cells using RNeasy Mini Kit (Qiagen 74104), as per manufacturer’s protocol. 1 ug RNA was used to synthesize cDNA in 10 µl volume by using RevertAid First Strand cDNA Synthesis Kit (Thermo scientific, K1621), as per as per manufacture’s protocol. To carry out RT-PCR, 1:100 diluted cDNA was used. The PCR (35 cycles) was carried out for SSTR2 using forward primer: 5′-GATGATCACCATGGCTGTG-3′and reverse primer: 5′-CAGGCATGATCCCTCTTC-3′; for SSTR5 using forward primer: 5′-GCCGGCCTCTACTTCTTCGTG-3′and reverse primer: 5′-CCGTGGCGTCAGCGTCCTTGG-3′; and for GAPDH using forward primer: 5′-CTTCATTGACCTCAACTACATGG-3′ and reverse primer: 5′-GTCTGGGTGGCAGTGATG-3′.

### Statistical analyses

All the graphs were generated and all the statistical analyses were done using Excel software (Microsoft Corporation). Comparisons were made using student’s *t*-test and *P*-values < 0.05 were considered significant.

## SUPPLEMENTARY MATERIALS FIGURES



## Abbreviations

BP: bronchopulmonary
DHQ: 1,5-dihydroxyisoquinoline
DSB: double strand break
DTPA: diethylenetriaminepentaacetic acid
GEP: gasrtroenteropancreatic
HRR: homologous recombination repair
NET: neuroendocrine tumor
PAR: polymer of ADP-ribose
PARP1: poly(ADP-ribose) polymerase 1
PARPi: PARP inhibitor
PRRT: peptide receptor radionuclide therapy
SSB: single strand break
SSTR: somatostatin receptor
