# Supplementary material for: Potentiation of 177Lu-octreotate peptide receptor radionuclide therapy of human neuroendocrine tumor cells by PARP inhibitor
Source: Oncotarget. 2018 May 15;9(37):24693–706. doi: 10.18632/oncotarget.25266 (PMC5973847; doi:10.18632/oncotarget.25266)
Supplement: Supplementary file 1 [file oncotarget-09-24693-s001.pdf]

Potentiation of ^{177}Lu-octreotate peptide receptor radionuclide therapy of human neuroendocrine tumor cells by PARP inhibitor

SUPPLEMENTARY MATERIALS

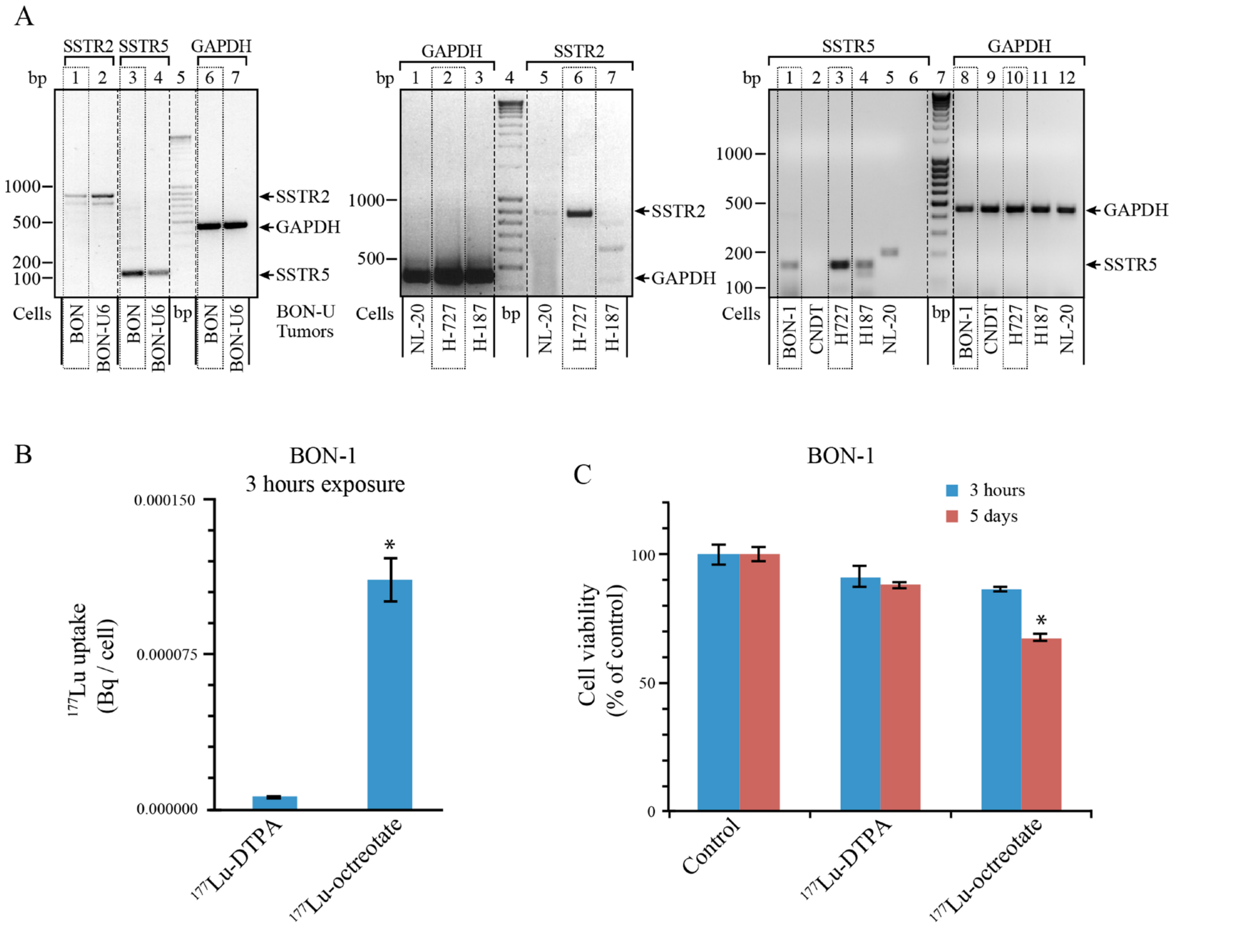

Supplementary Figure 1: (**A**) RT-PCR for expression of SSRT2 and SSTR5 receptors. The expression levels of SSTR2 and 5 in indicated NET and non-NET cells were analyzed by RT-PCR. GAPDH is the loading control. (**B**) Uptake of 177Lu-octreotate in BON-1 cells. The cells were exposed to 2.75 MBq/mL of ^{177}Lu-octreotate or 2.75 MBq/mL of ^{177}Lu-DTPA for 3 hours. Each data point, derived from six replicates per experimental condition, represents mean ± SEM. (**C**) Comparison of 177Lu-octreotate- and ^{177}Lu-DTPA-induced reduction in cell viability of BON-1 cells after a 3-hour treatment with that after a 5-day treatment. The cells were exposed to 2.75 MBq/mL of ^{177}Lu-octreotate or 2.75 MBq/mL of ^{177}Lu-DTPA for 3 hours and 5 days. The cell count in each treatment group is expressed as percent of number of viable cells in untreated control. The average of at least five replicates per experimental condition is plotted as mean ± SEM. After 3 hours of exposure of BON-1 cells to these treatments, while there was a significantly higher level of internalization of ^{177}Lu-octreotate as compared to the ^{177}Lu-DTPA (B), there was no difference in toxicity induced by ^{177}Lu-octreotate or ^{177}Lu-DTPA as compared to untreated control (C; blue bars). We observed a significant increase in toxicity induced by ^{177}Lu-octreotate as compared to ^{177}Lu-DTPA in BON-1 cells when the exposure time was increased to 5 days (C; red bars). This difference in toxicity could be attributed to the specific uptake and intracellular retention of ^{177}Lu-octreotate, and not of ^{177}Lu-DTPA, via SSTR during the 5 days of treatment in BON-1 cells. Therefore, we concluded that the treatment of BON-1 cells with ^{177}Lu-octreotate for 5 days is a better time point to observe a moderate toxicity (e.g. that could be enhanced by PARP inhibition) induced by ^{177}Lu-octreotate due to its specific internalization in the BON-1 cells over that due to the non-internalized radioactivity in the medium. * indicates a significant difference ($P \leq 0.05$) in % viability of cells treated with ^{177}Lu-octreotate for 3 h and 5 days.

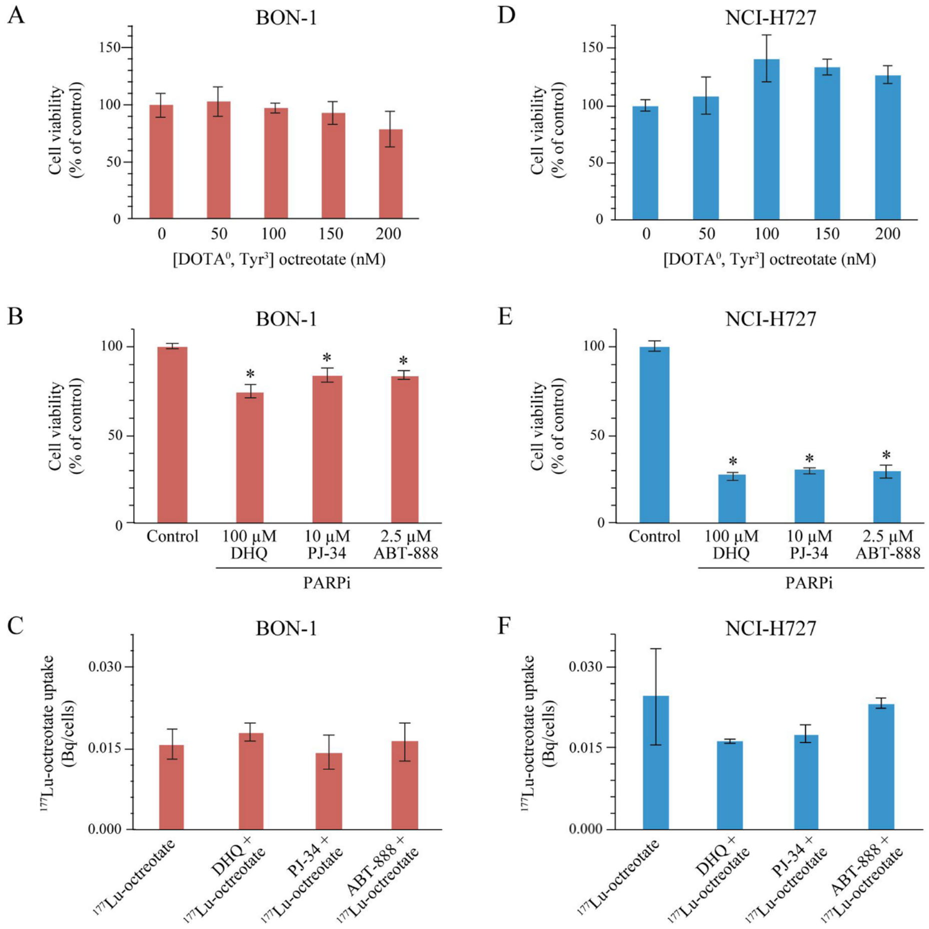

Supplementary Figure 2: (**A**) Toxicity of unlabeled [DOTA^0-Tyr^3]-octreotate in 2D model of BON-1 cells. The cells were treated with increasing concentrations of unlabeled [DOTA^0-Tyr^3]-octreotate for 5 days followed by another 5 days incubation in the medium without the unlabeled [DOTA^0-Tyr^3]-octreotate. The cell counts in each treatment groups are expressed as percent of number of cells in untreated control. The average of six replicates per experimental condition are plotted as mean ± SEM. There was no significant difference between each of the data points. (**B**) Toxicity of three different PARPis in BON-1 cells. The cells were treated with three different PARPi for 10 days. The cell counts are expressed as percent of number of cells in untreated control and the average of six replicates per experimental condition was plotted as mean ± SEM. * indicates a significant difference ($P < 0.05$) relative to the untreated controls. (**C**) Effect of three different PARPis on the incorporation of ^{177}Lu-octreotate in 2D model of BON-1 cells. Cells were treated with 2.75 MBq/mL ^{177}Lu-octreotate in presence or absence of PARPi for 5 days. Each data point, derived from triplicates per experimental condition, represents mean ± SD. There was no significant difference between each of the data points. (**D**) Toxicity of unlabeled [DOTA^0-Tyr^3]-octreotate in 2D model of H727 cells. The cells were treated as described in panel A. The cell count in each treatment group is expressed as percent of number of cells in untreated control. The average of six replicates per experimental condition is plotted as mean ± SEM. There was no significant difference between each of the data points. (**E**) Toxicity of three different PARPis in H727 cells. The cells were treated as described in panel B. The cell counts are expressed as percent of number of cells in untreated control and the average of six replicates per experimental condition is plotted as mean ± SEM. *indicates a significant difference ($P < 0.05$) relative to the untreated controls. (**F**) Effect of three different PARPis on the incorporation of ^{177}Lu-octreotate in 2D model of H727 cells. Cells were seeded and treated as described for BON-1 cells in panel C and were processed for ^{177}Lu-octreotate uptake measurements. Each data point, derived from triplicates per experimental condition, represents mean ± SD. There was no significant difference between each of the data points.

A

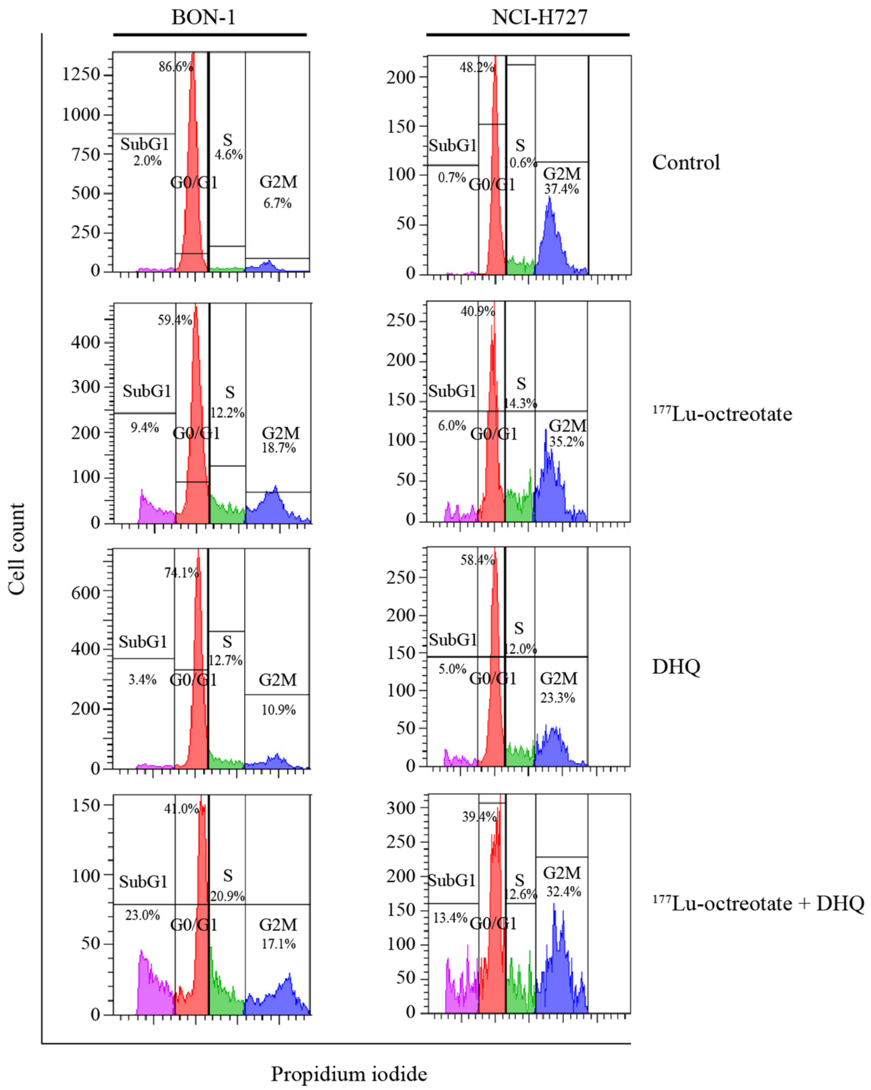

B

BON-1 cells				
Treatment	Average % cell population ± S.D.			
	% SubG1	% G0/G1	% S	% G2/M
Untreated	2.0 ± 1.2	86.6 ± 2.5	4.6 ± 0.5	6.7 ± 0.7
^{177}Lu-octreotate	9.4 ± 2.5	59.4 ± 5.2	12.2 ± 0.7	18.7 ± 2.3
DHQ	3.4 ± 1.3	74.1 ± 3.3	12.7 ± 1.9	10.9 ± 0.4
^{177}Lu-octreotate +DHQ	23.0 ± 1.2	41.9 ± 1.3	20.9 ± 4.1	17.1 ± 2.0

NCI-H727 cells				
Treatment	Average % cell population ± S.D.			
	% SubG1	% G0/G1	% S	% G2/M
Untreated	0.7 ± 0.1	48.2 ± 2.4	10.6 ± 0.3	37.4 ± 1.9
^{177}Lu-octreotate	6.0 ± 0.6	40.9 ± 1.2	14.3 ± 1.1	35.2 ± 2.8
DHQ	5.0 ± 1.1	58.4 ± 2.9	12.0 ± 0.7	23.3 ± 1.6
^{177}Lu-octreotate +DHQ	13.4 ± 0.6	39.4 ± 0.9	12.6 ± 0.9	32.4 ± 0.7

Supplementary Figure 3: (**A**) Histograms of cell cycle profile of BON-1 and NCI-H72. BON-1 (left panel) and NCI-H727 (right panel) cells were treated in triplicate as described in Figure 2B and Figure 4B and these histograms were created using FlowJo 7.6.1 software from Tree Star. (**B**) Table of average % cell population in each phase of cell cycle in response to different treatments of BON-1 and H727 cells.

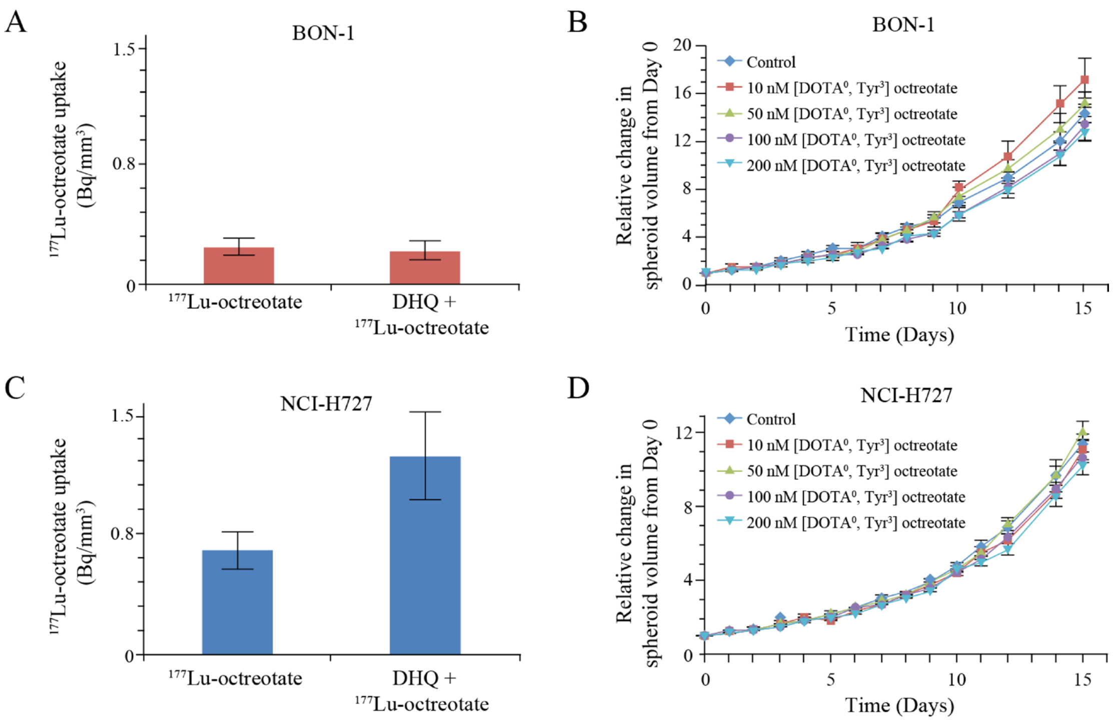

Supplementary Figure 4: (**A**) ^{177}Lu-octreotate uptake by BON-1 spheroids. Spheroids were treated with 2.75 MBq/mL ^{177}Lu-octreotate in presence or absence of PARPi for 5 days and 6 to 12 spheroids per treatment group were processed for measurement of ^{177}Lu-octreotate uptake. Mean ± SEM of Bq per mm^3 of spheroids is plotted. (**B**) Effect of unlabeled [DOTA0-Tyr3]-octreotate in BON-1 spheroids. Spheroids were treated with 50–200 nM [DOTA^0-Tyr^3]-octreotate, and their growth was monitored and expressed as mean ± SEM of fold change in the spheroid volume relative to that at the start of treatment, i.e. Day 0. (**C**) ^{177}Lu-octreotate uptake by H727 spheroids. Spheroids were treated with 2.75 MBq/mL ^{177}Lu-octreotate in presence or absence of PARPi for 5 days and 6 to 12 spheroids per treatment group were processed for measurement of ^{177}Lu-octreotate
